# Supplementary figures and images for: Geographic range size and species morphology determines the organization of sponge host-guest interaction networks across tropical coral reefs
Source: PeerJ. 2023 Nov 24;11:e16381. doi: 10.7717/peerj.16381 (PMC10680448; doi:10.7717/peerj.16381)

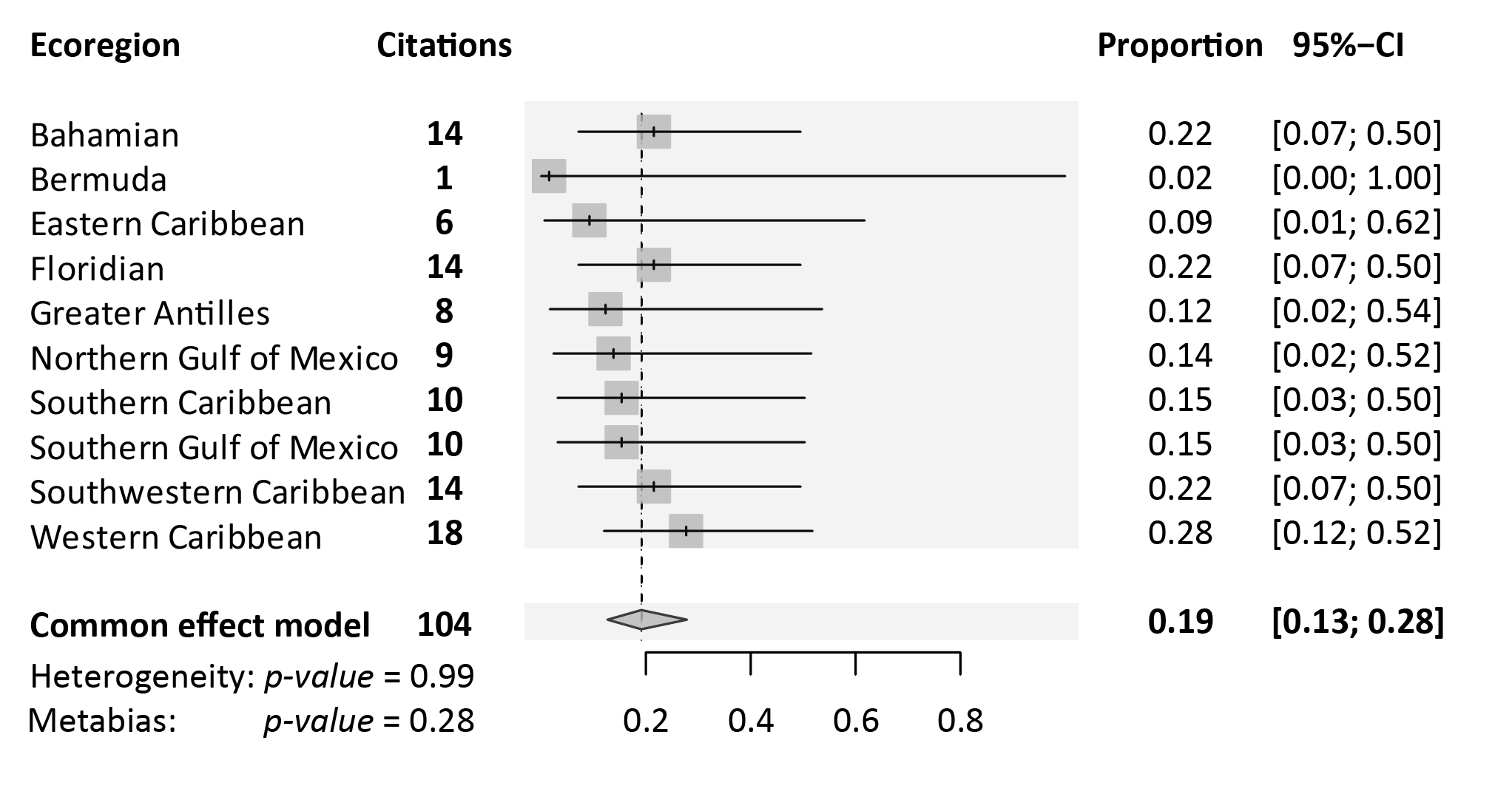

Supplement: Supplemental Information 1 — This graph exhibits the number of citations per EcoRegion and their corresponding proportion. Neither Citation Heterogeneity nor Citation Bias are statistically significant. The ecoregions are based on the Marine Ecoregions of the World classification (Spalding et al., 2007) [file peerj-11-16381-s001.png]

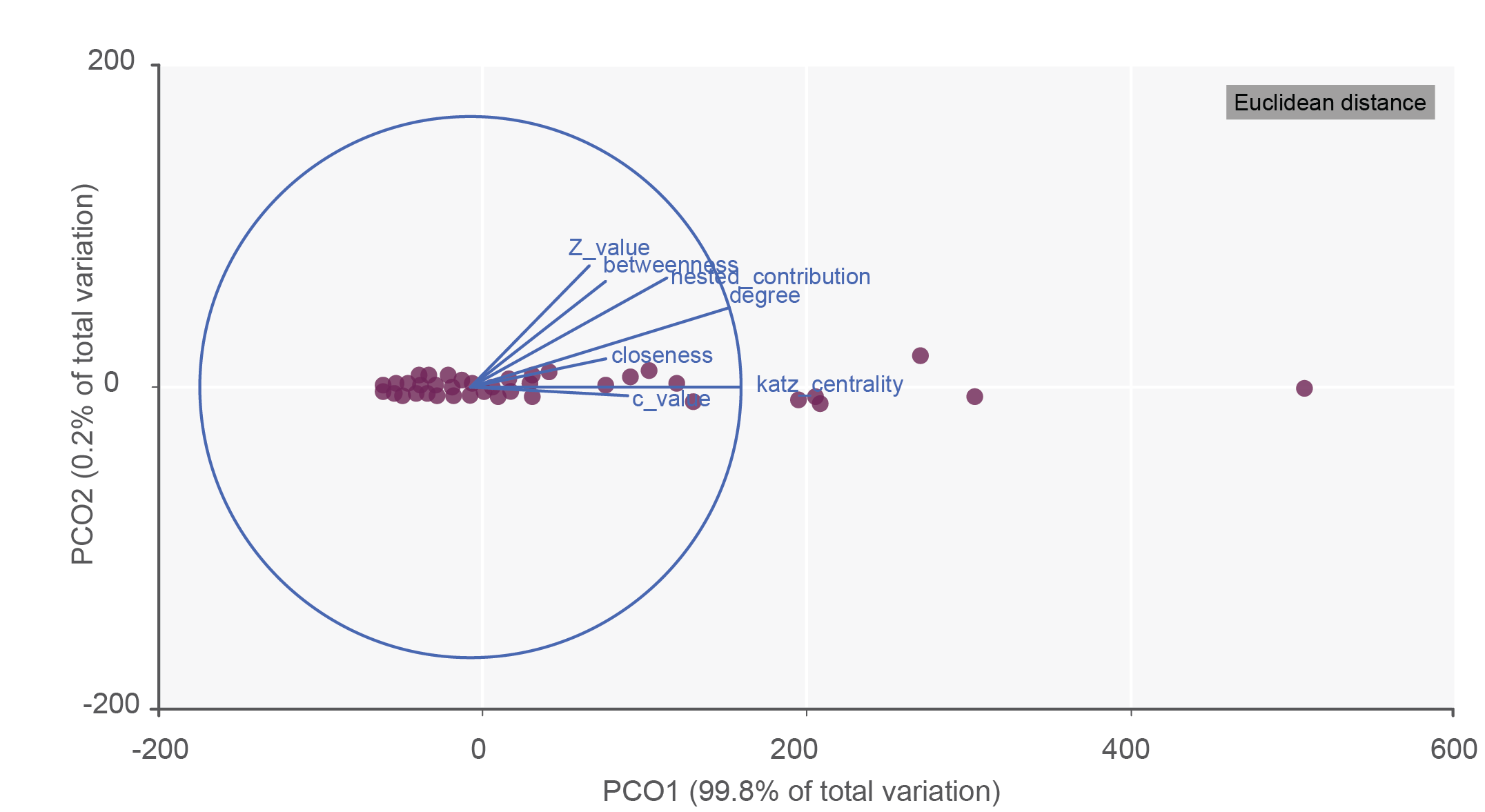

Supplement: Supplemental Information 2 — Purple dots represents each sponge species, and the similarity between sponges (Euclidean distance) are calculated according nine network centrality index (species-level descriptors): species degree, betweenness, closeness, Katz centrality, among-module connectivity (C_value), standardized within-module degree (Z_value), and nestedness contribution. We use Pearson’s correlation for the species-level descriptors. [file peerj-11-16381-s002.png]
